# Gender and Age Impact on the Association Between Thyroid-Stimulating Hormone and Serum Lipids

**DOI:** 10.1097/MD.0000000000002186

**Published:** 2015-12-11

**Authors:** Zhaowei Meng, Ming Liu, Qing Zhang, Li Liu, Kun Song, Jian Tan, Qiang Jia, Guizhi Zhang, Renfei Wang, Yajing He, Xiaojun Ren, Mei Zhu, Qing He, Shen Wang, Xue Li, Wei Zheng, Tianpeng Hu, Na Liu, Arun Upadhyaya, Pingping Zhou, Jianping Zhang

**Affiliations:** From the Department of Nuclear Medicine (ZM, JT, QJ, GZ, RW, YH, SW, XL, WZ, TH, NL, AU, PZ), Department of Endocrinology and Metabolism (ML, XR, MZ, QH), Department of Health Management, Tianjin Medical University General Hospital (QZ, LL, KS), and Department of Nuclear Medicine, Tianjin Third Central Hospital, Tianjin, P.R. China (JZ).

## Abstract

The relationship between thyroid-stimulating hormone (TSH) and hyperlipidemia is still a topic of debate. We aimed to explore the impact of gender and age on the association between serum TSH and lipid profile in a large cohort of Chinese.

This cross-sectional study enrolled 13,915 participants (8565 male, 5350 female), who self-reported as healthy without any known previous diseases. Clinical data including anthropometric measurements, thyroid function, and other serum parameters were collected. The associations between TSH and hyperlipidemia of males and females were analyzed separately after dividing TSH and age into subgroups. Odds ratio for hyperlipidemia was calculated by binary logistic regression models.

Young males had significantly higher prevalence of hypercholesterolemia, hypertriglyceridemia, and high serum low-density lipoprotein-cholesterol than females, yet after menopause, females had higher prevalence than males. TSH was positively associated with hyperlipidemia independent of thyroid hormones. Males showed more reduced risks of hyperlipidemia in low TSH concentrations, while females demonstrated more enhanced risks of hyperlipidemia in high TSH concentrations. For instance, if TSH was lower than 0.3 μIU/mL, the risks of developing hypercholesterolemia and hypertriglyceridemia in males were only 0.198 (*P* < 0.01) and 0.425 (*P* < 0.05) of the reference TSH risks (between 2.0 and 3.0 μIU/mL), while in females the risks were 0.553 (*P* < 0.05) and 0.642 (*P* > 0.05), respectively. If TSH was higher than 4.0 μIU/mL, women displayed significantly higher risks of developing hypertriglyceridemia than the reference TSH risks (*P* < 0.05), yet, men did not demonstrate such significances.

Our results showed thyroid hormone independent positive associations between serum TSH and lipids, which were substantially influenced by gender and age. Males demonstrated more protective effects of low TSH against hyperlipidemia, while females showed more detrimental effects of high TSH on hyperlipidemia.

## INTRODUCTION

The world faces a burden of thyroid disease that has reached epidemic proportions. It is estimated that around 200 million individuals worldwide have various kinds of thyroid dysfunctions.^1^ The association between thyroid function and serum lipid status has become a popular area of research in recent years. It is well recognized that thyroid function can influence the synthesis, mobilization, and degradation of lipids.^2,3^ Recent studies have indicated that thyroid-stimulating hormone (TSH) is associated with adverse changes of lipid metabolism and increased cardiovascular risks as well.^3–11^ For instance, Xu et al^4^ proved that TSH can increase total cholesterol (TC) level independent of thyroid hormones. Several other studies did not observe such an association,^12–15^ leading to a controversy. For example, Rodondi et al^12^ indicated that subclinical hypothyroidism was not associated with increased risk for coronary heart disease, stroke, peripheral arterial disease, or cardiovascular-related or total mortality. Therefore, confirmative studies with large recruited subjects are needed.

Gender and age can influence the relationship between thyroid function and lipid profiles.^16^ Yet, on the one hand, this aspect has not been thoroughly investigated; on the other, the available literature is still controversial.^16–18^ For example, by using stepwise regression analysis, Tognini et al^16^ revealed that age was the most crucial factor influencing serum cholesterols, and TSH was the second important factor following age. Furthermore, this research also found that both age and TSH could influence either TC or low-density lipoprotein-cholesterol (LDL) significantly in females. Yet, in males, TSH only strongly affected TC. Nevertheless, in males, both TSH and body mass index (BMI) greatly affected triglycerides (TG) concentrations independently of age, while in women, TSH did not show any obvious influence on TG. The Tromsø study,^17^ which recruited 5143 participants, displayed a significantly positive correlation between TC and TSH, as well as LDL and TSH, in both sex. However, in women, if adjustment was made by age and BMI, these relationships became not significant. Therefore, these discordant results warrant a large-scale investigation with the emphasis on both age and gender.

The aim of the current study was to evaluate the impact of gender and age on the association between TSH and lipid profile and the burden of dyslipidemia in a large cross-sectional Han Chinese population sample.

## METHODS

### Design

This cross-sectional study was conducted in Tianjin Medical University General Hospital in China, under collaboration from the departments of Health Management, Endocrinology & Metabolism, and Nuclear Medicine. During the period from September 2011 to April 2014, a total of 13,915 eligible subjects (8565 male, 5350 female) participated in this mainly community-based health check program. This work was a part of our continuous project for the general public health of Tianjin Municipality, and the protocol was developed and executed previously by our group.^19–21^ All participants were asked to complete a self-reported questionnaire and provide an overnight fasting blood sample. All participants were self-reported as healthy without any known previous diseases. In order to avoid the influence of confounding factors, the following criteria were used for exclusion: subjects with thyroid, hepatic, renal, gastro-intestinal diseases, or oncology; subjects with any diseases or taking any medicine that might affect their thyroid status or lipid metabolism (eg, antithyroid drugs, thyroid hormone, amiodarone, iodine, estrogen, androgen, statins, steroid hormones, etc.); and pregnancy. Written consents were obtained, and the institutional review board and ethic committee of Tianjin Medical University General Hospital approved this study.

### Measurements

Anthropometric measurements and fasting blood tests of the participants were performed during their visits to our institution. Body height and body weight were measured in centimeters and kilograms. BMI was calculated by dividing body weight (kg) by the square of body height ().^2^ Fasting blood samples were obtained between 7 am and 10 am. TSH, free triiodothyronine (FT3), and free thyroxine (FT4) were analyzed on a fully automated ADVIA Centaur analyzer (Siemens Healthcare Diagnostics, Erlangen, Germany) by chemiluminescent reaction principle. TC, TG, LDL, HDL, alanine aminotransferase, total bilirubin, blood urea nitrogen, uric acid, creatinine (Cr), and fasting glucose were determined enzymatically by an auto-analyzer (Hitachi Model 7600 analyzer, Hitachi, Tokyo, Japan).

### Definition

The diagnostic criteria for dyslipidemia were in accordance with the National Cholesterol Education Program Adult Treatment Panel III criteria as follows: TC ≥ 5.18 mmol/L, TG ≥ 1.70 mmol/L, LDL ≥ 3.37 mmol/L, and HDL < 1.04 mmol/L.^22^ Participants were divided into subgroup 1 to 8 according to TSH levels: TSH < 0.3 μIU/mL, 0.3 μIU/mL ≤ TSH < 1.0 μIU/mL, 1.0 μIU/mL ≤ TSH < 2.0 μIU/mL, 2.0 μIU/mL ≤ TSH < 3.0 μIU/mL, 3.0 μIU/mL ≤ TSH < 4.0 μIU/mL, 4.0 μIU/mL ≤ TSH < 5.0 μIU/mL, 5.0 μIU/mL ≤ TSH < 10.0 μIU/mL, and TSH ≥ 10.0 μIU/mL. Participants were also divided into subgroup 1 to 7 based on ages: age < 25 years, 25 years ≤ age < 35 years, 35 years ≤ age < 45 years, 45 years ≤ age < 55 years, 55 years ≤ age < 65 years, 65 years ≤ age < 75 years, and age ≥ 75 years.

### Statistical Analysis

Statistics were done as our prior protocols.^19–21^ All data were presented as mean ± standard deviation. Differences of indices between groups were analyzed by independent sample's *t*-test or one-way analysis of variance. For multiple comparisons among different subgroups, the least significant difference test was applied. In order to compare differences of intergroup prevalence, Chi-square test was performed. Pearson bivariate correlation was conducted between TSH and other variables. After data stratification according to different thyroid functional states, binary logistic regression models were executed to calculate odds ratio for hyperlipidemia with 95% confidence interval (CI). Statistical Package for Social Sciences (SPSS version 17.0, Chicago, IL) was used for statistics. And a *P* value of <0.05 was defined as significance.

## RESULTS

### The Characteristics of the Participants in Different Gender

The characteristics of the participants were summarized (Table 1). There were significant differences in all parameters with respect to opposite gender. Males were younger than females, yet BMI in males was higher than in females. TSH was lower in males than in females, while FT3 and FT4 were higher in males than in females. Serum lipids, hepatic, and renal functions were also different between genders.

**TABLE 1:**
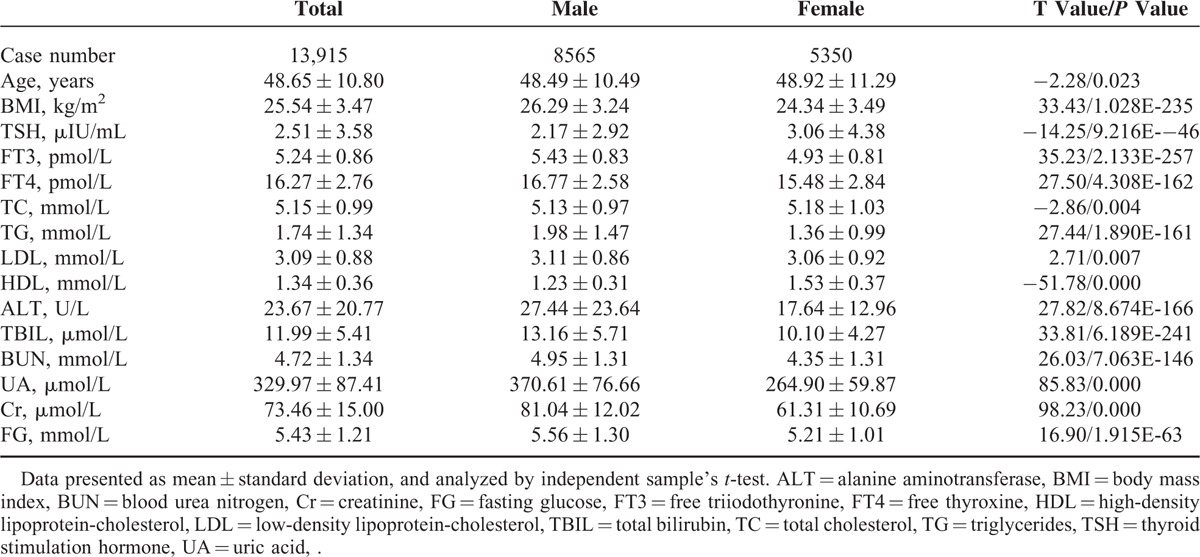
Population Characteristics Based on Different Genders

**FIGURE 1:**
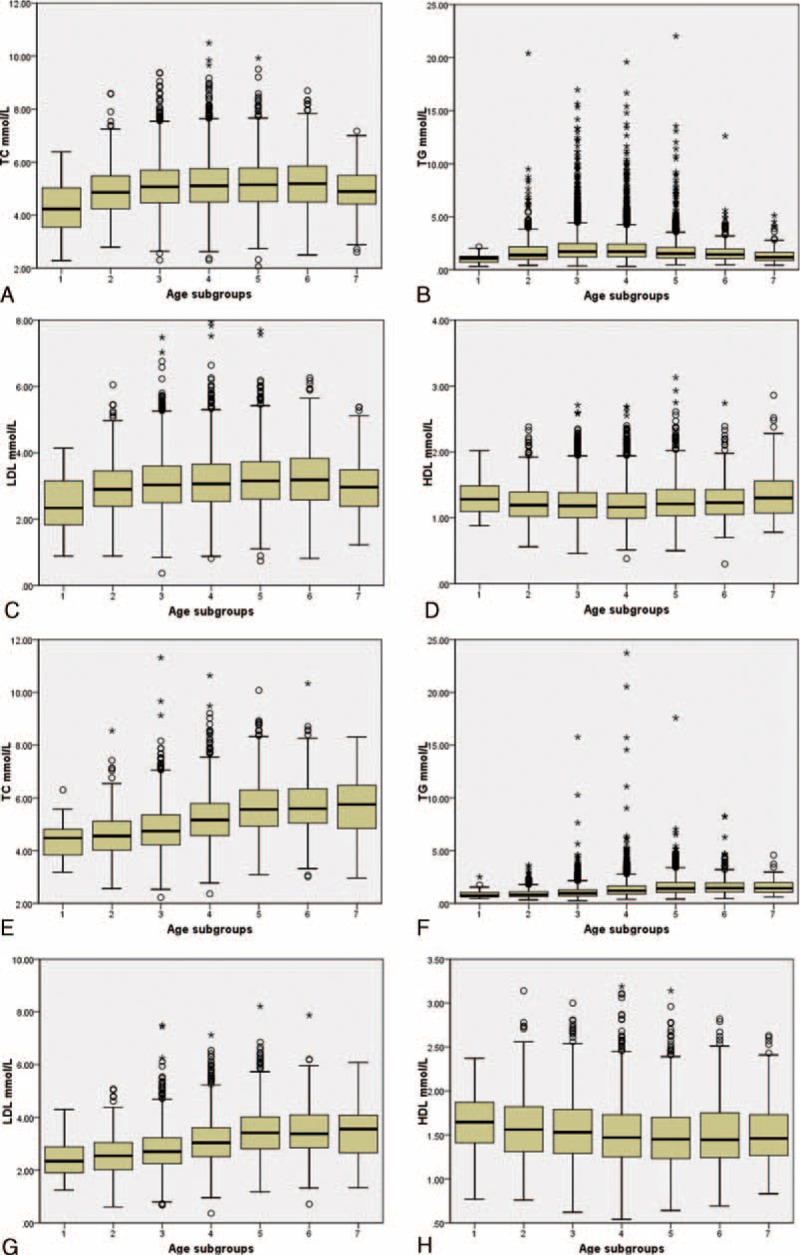
Serum lipid distribution in different age subgroups. Box plots were drawn based on different age subgroups in males (A–D) and in females (E–H). Age subgroups 1 to 7 referred to the followings: age < 25 years, 25 years ≤ age < 35 years, 35 years ≤ age < 45 years, 45 years ≤ age < 55 years, 55 years ≤ age < 65 years, 65 years ≤ age < 75 years, age ≥ 75 years. HDL = high-density lipoprotein-cholesterol, LDL = low-density lipoprotein-cholesterol, TC = total cholesterol, TG = triglycerides.

Age cast differences among the participants (Fig. 1). For males, TC, TG, and LDL showed the tendency of increase from the youngest to the age range of 65 to 75, and then decreased in the eldest participants. Inversely, HDL demonstrated a decrease trend. For females, TC, TG, and LDL showed constant trend of increase from the youngest till the eldest, and HDL displayed constant decrease trend.

**FIGURE 2:**
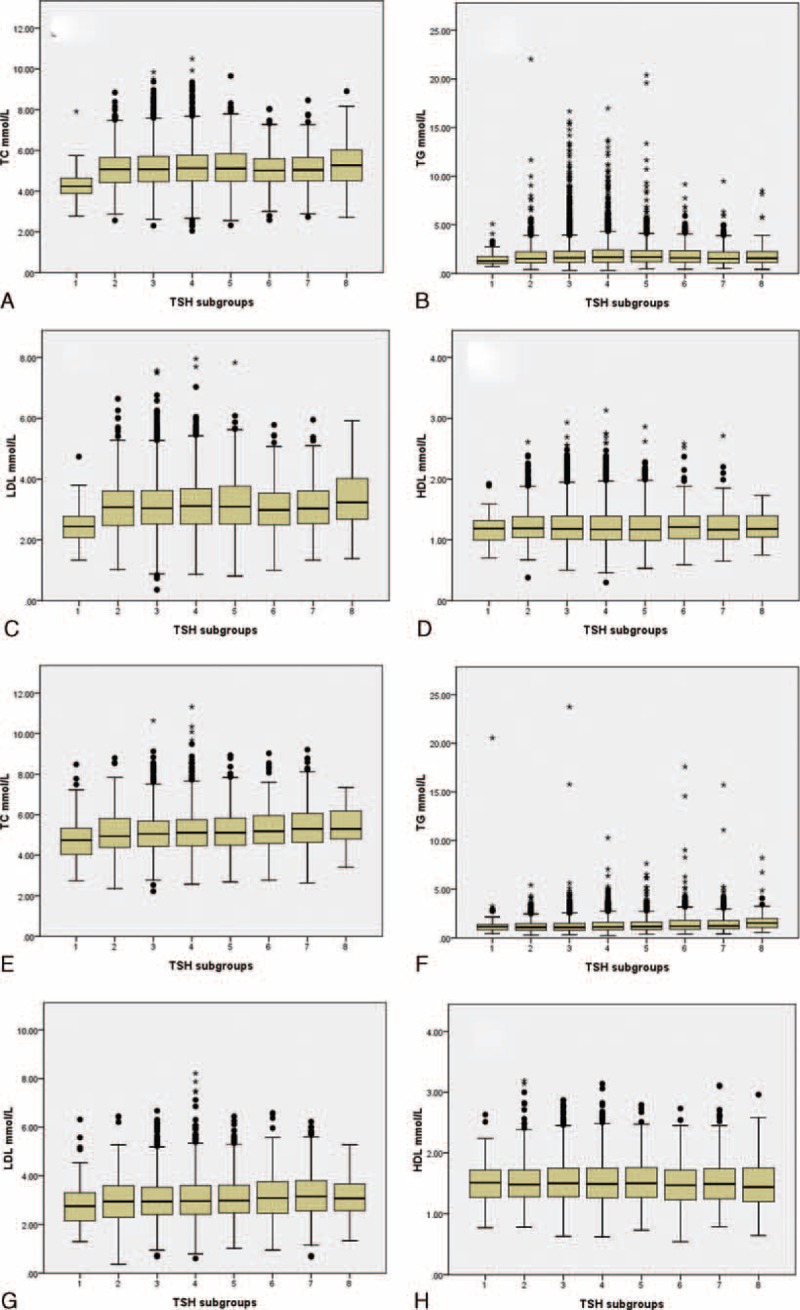
Serum lipid distribution in different TSH subgroups. Box plots were drawn based on different TSH subgroups in males (A–D) and in females (E–H). TSH subgroups 1 to 8 referred to the followings: TSH < 0.3 μIU/mL, 0.3 μIU/mL ≤ TSH < 1.0 μIU/mL, 1.0 μIU/mL ≤ TSH < 2.0 μIU/mL, 2.0 μIU/mL ≤ TSH < 3.0 μIU/mL, 3.0 μIU/mL ≤ TSH < 4.0 μIU/mL, 4.0 μIU/mL ≤ TSH < 5.0 μIU/mL, 5.0 μIU/mL ≤ TSH < 10.0 μIU/mL, TSH ≥ 10.0 μIU/mL. HDL = high-density lipoprotein-cholesterol, LDL = low-density lipoprotein-cholesterol, TC = total cholesterol, TG = triglycerides, TSH = thyroid stimulating hormone.

The differences of serum lipids based on TSH levels were demonstrated in Figure 2. TC in both sex, TG in female, as well as LDL in male, showed consistent increase from the lowest TSH level to the highest TSH level. The differences of HDL levels among the TSH subgroups were relatively subtler in both genders.

### Prevalence of Hyperlipidemia in Different Gender

**FIGURE 3:**
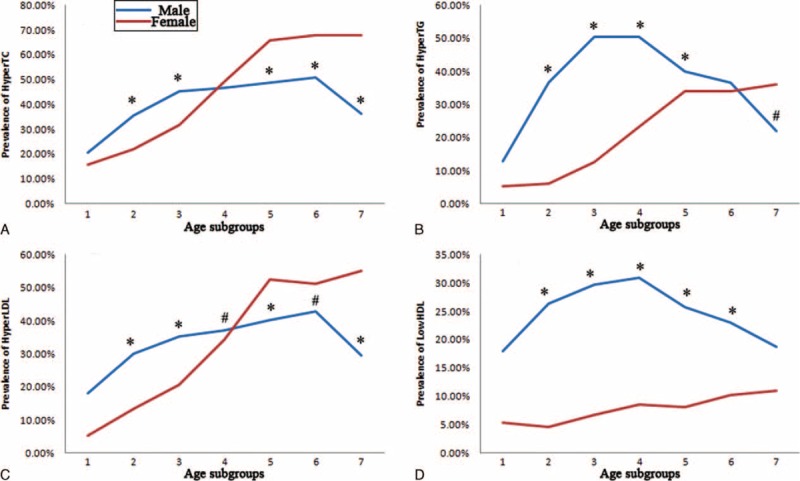
Prevalence of dyslipidemia in different age subgroups. Definition of dyslipidemia: hypercholesterolemia (HyperTC), TC ≥ 5.18 mmol/L; hypertriglyceridemia (HyperTG), TG ≥ 1.70 mmol/L; high serum level of low-density lipoprotein-cholesterol (HyperLDL), LDL ≥ 3.37 mmol/L; low serum level of high-density lipoprotein-cholesterol (LowHDL), HDL < 1.04 mmol/L. Age subgroups 1 to 7 referred to the followings: age < 25 years, 25 years ≤ age < 35 years, 35 years ≤ age < 45 years, 45 years ≤ age < 55 years, 55 years ≤ age < 65 years, 65 years ≤ age < 75 years, age ≥ 75 years. HDL = high-density lipoprotein-cholesterol, LDL = low-density lipoprotein-cholesterol, TC = total cholesterol, TG = triglycerides. ^#^Difference of prevalence between gender was significant at 0.05; ^∗^Difference of prevalence between gender was significant at 0.01.

The prevalence of hypercholesterolemia and high serum LDL showed the same increasing tendency in males from the youngest to the age range of 65 to 75, and then decreased in the eldest participants. Yet, in females, this prevalence tendency increased from the youngest to the eldest (Fig. 3). Young males (younger than 45) had significantly higher prevalence than females. However, after menopause (older than 55), females had significantly higher prevalence than males. This crisscross pattern was also discovered in hypertriglyceridemia, yet the converging point was around 65 to 75 years of age. For low-serum HDL, males always showed higher prevalence than females indiscriminate of age.

**FIGURE 4:**
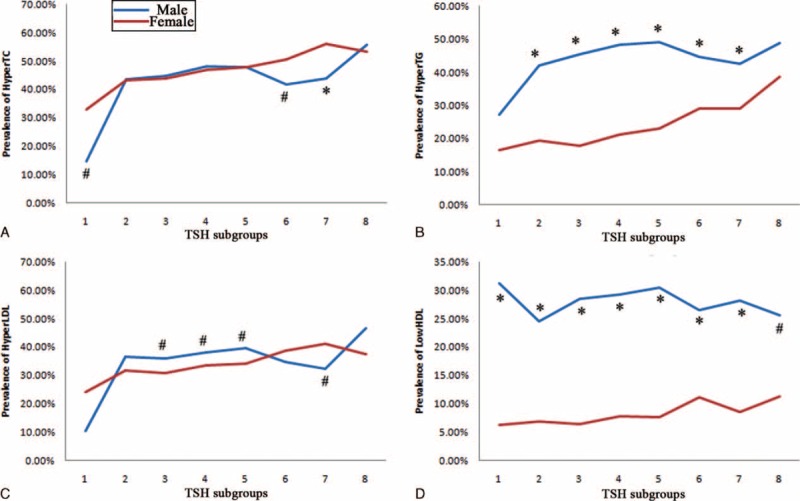
Prevalence of dyslipidemia in different TSH subgroups. Definition of dyslipidemia: hypercholesterolemia (HyperTC), TC ≥ 5.18 mmol/L; hypertriglyceridemia (HyperTG), TG ≥ 1.70 mmol/L; high serum level of low-density lipoprotein-cholesterol (HyperLDL), LDL ≥ 3.37 mmol/L; low serum level of high-density lipoprotein-cholesterol (LowHDL), HDL < 1.04 mmol/L. TSH subgroups 1 to 8 referred to the followings: TSH < 0.3 μIU/mL, 0.3 μIU/mL ≤ TSH < 1.0 μIU/mL, 1.0 μIU/mL ≤ TSH < 2.0 μIU/mL, 2.0 μIU/mL ≤ TSH < 3.0 μIU/mL, 3.0 μIU/mL ≤ TSH < 4.0 μIU/mL, 4.0 μIU/mL ≤ TSH < 5.0 μIU/mL, 5.0 μIU/mL ≤ TSH < 10.0 μIU/mL, TSH ≥ 10.0 μIU/mL. HDL = high-density lipoprotein-cholesterol LDL = low-density lipoprotein-cholesterol, TC = total cholesterol, TG = triglycerides, TSH = thyroid stimulating hormone. ^#^Difference of prevalence between gender was significant at 0.05; ^∗^Difference of prevalence between gender was significant at 0.01.

Prevalence of hyperlipidemia rose when TSH concentration rose, yet no crisscross pattern was found (Fig. 4). The prevalence of hypercholesterolemia was higher in females if TSH was less than 0.3 μIU/mL, or between 4.0 and 10.0 μIU/mL, otherwise no significant differences existed. The prevalence of high serum LDL was higher in females if TSH was between 5.0 and 10.0 μIU/mL, this prevalence was higher in males if TSH was between 1.0 and 4.0 μIU/mL. For the prevalence of hypertriglyceridemia and low-serum HDL, males always showed higher prevalence than females.

### Incidence of Thyroid Dysfunction in Different Gender

Females had significantly higher thyroid dysfunction incidence than males (Table 2), hypothyroidism and hyperthyroidism were defined as TSH > 5.0 μIU/mL and TSH < 0.3 μIU/mL, respectively. Most age subgroups demonstrated the same pattern of differences in opposite sex, except for the youngest and the eldest. There existed a significant increasing tendency of hypothyroidism incidence with aging (except for the youngest) for both gender (Chi value for male, 86.911, *P* = 1.326E-16; Chi value for female 58.561; *P* = 8.818E-11).

**TABLE 2:**
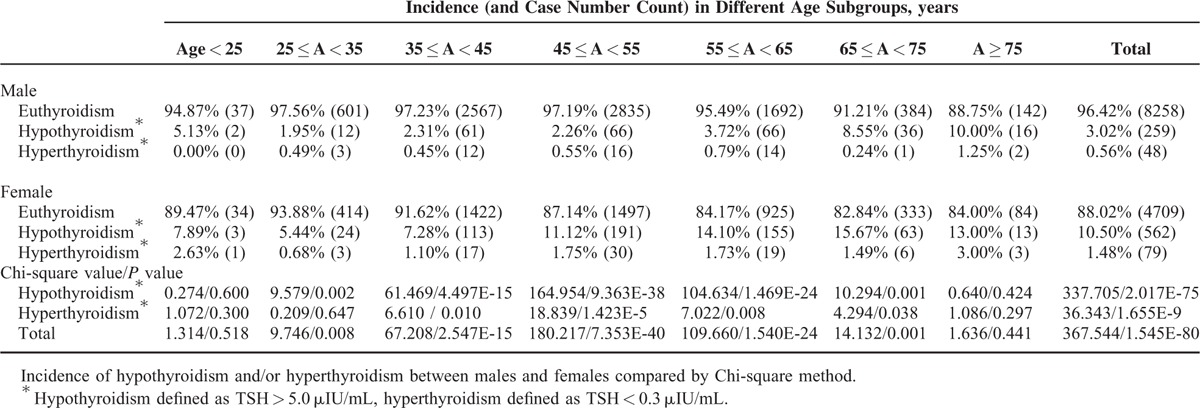
Incidence of Hypothyroidism and Hyperthyroidism on Different Genders

### Correlations Between TSH and Serum Lipid Levels in Different Gender

Significant negative relationships were demonstrated for FT3 and FT4 for both genders. In males, positive relationships were demonstrated for age, TC, LDL, and Cr. In females, positive relationships were demonstrated for age, BMI, TC, TG, LDL, alanine aminotransferase, blood urea nitrogen, uric acid, and Cr (Table 3).

**TABLE 3:**
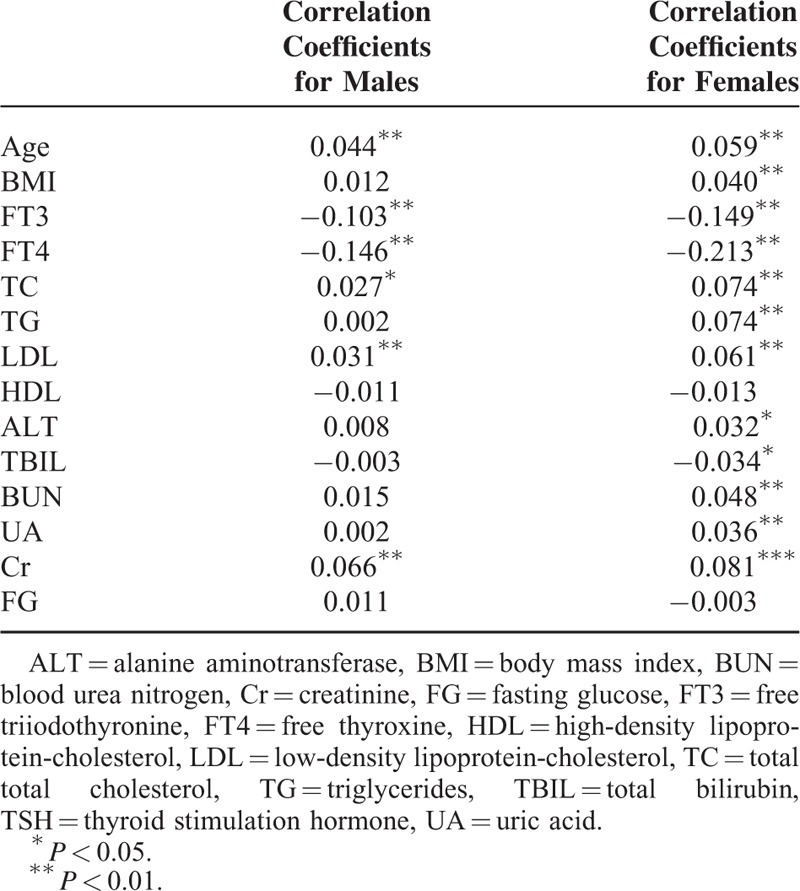
Pearson Bivariate Correlations Between TSH and Other Variables Based on Different Genders

### Risks of Developing Hyperlipidemia in Different TSH Concentrations in Opposite Gender

Binary logistic regression models were utilized on different thyroid functional state. Thyroid functional states were designated as the categorical variables, and the mid-normal TSH group of “2.0 μIU/mL ≤ TSH < 3.0 μIU/mL” was set as the reference. Model 1 had FT3 and FT4 as covariates. Model 2 had all parameters as covariates (Tables 4 and 5). In males, we demonstrated significantly reduced risks of hyperlipidemia while TSH decreased from the reference level, indicating protective effects of low TSH against hyperlipidemia. For instance, in Model 2, the risks of hypercholesterolemia, hypertriglyceridemia, and high serum level of LDL in TSH subgroup 1 were only 0.198 (*P* < 0.01), 0.425 (*P* < 0.05), and 0.219 (*P* < 0.01) of the corresponding reference TSH subgroup risks. In females, the reduced risks of hyperlipidemia along with the decreasing TSH level did not reach such significance as in males. For example, in Model 2, the risks of hypercholesterolemia, hypertriglyceridemia, and high serum level of LDL were just 0.553 (*P* < 0.05), 0.642 (*P* > 0.05), and 0.635 (*P* > 0.05) of the reference risks. On the other hand, our results displayed that if TSH level rose, the risks of hyperlipidemia generally increased, especially in the subgroup of TSH ≥ 10.0 μIU/mL, indicating detrimental effects of high TSH on hyperlipidemia. However, this pattern appeared to be much more significant in females than in males, particularly for hypertriglyceridemia. In females, if TSH was higher than 4.0 μIU/mL, risks of developing hypertriglyceridemia were significantly higher in both models than the reference TSH subgroup risks (all *P* < 0.05). Nevertheless, in males so such significances existed.

**TABLE 4:**
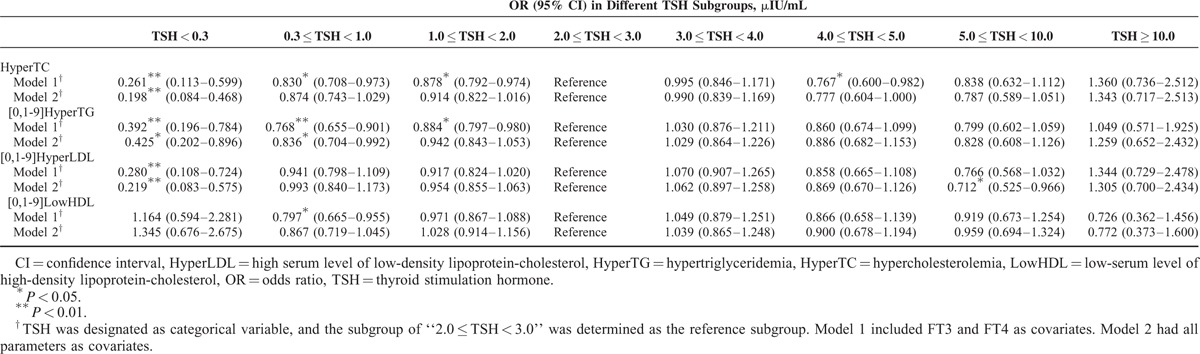
The Likelihood of Hyperlipidemia Among Different Thyroid Functional States in Logistic Regression Models for Males

**TABLE 5:**
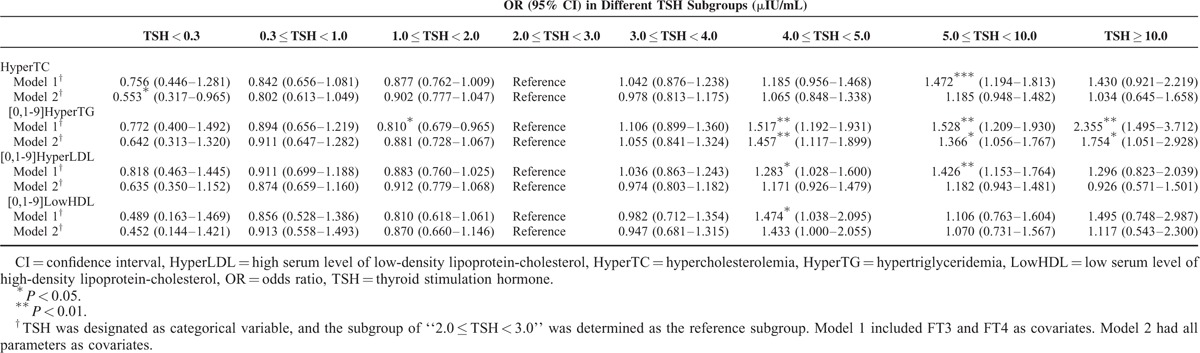
The Likelihood of Hyperlipidemia Among Different Thyroid Functional States in Logistic Regression Models for Females

## DISCUSSION

Clinical and subclinical thyroid dysfunctions are both very common clinical entities, the latter usually denotes the presence of a disease without obvious symptoms.^1^ Mild thyroid dysfunction often means the evolution of the disease at an early stage.^1^ The incidence of mild hypothyroidism and mild hyperthyroidism were reported to be around 9.5%^11^ and 1.8%,^23^ respectively. Results of the current study not only were in concord with the above-reported data, we also distinguished incidence disparity between genders. Mild hypothyroidism represents early thyroid failure, which is usually progressive, especially when TSH is higher than 10 μIU/mL, in females and with positive thyroid peroxidase antibodies.^24,25^ Hypothyroidism is associated with greater cardiovascular risk factors, including hyperlipidemia,^1,11,26,27^ making it an important topic in people's health.

Recently, a large number of clinical studies have indicated that TSH is associated with a deleterious change of lipid metabolism. And it has been suggested that the proatherogenic influence of TSH on serum lipid profile is in a thyroid hormone independent manner. Abnormal elevation of TC, LDL, and TG, and decrease of HDL have been observed in patients with subclinical hypothyroidism,^5,6^ or even in subject with normal TSH (TSH near the upper limit within the normal reference range).^6–10^ The current large population-based study was able to indicate protective effects of low-TSH concentration against hyperlipidemia and enhanced risks of hyperlipidemia in high-TSH concentration; these effects were independent of thyroid hormones. Our results were in line with the above-mentioned reports, confirming a linear and significant increase of hyperlipidemia with elevation of serum TSH levels.^4,7^ The exact mechanism for the direct effects of TSH on lipids has not been fully elucidated with sensible explanations. Only some plausible hypotheses were put forward as followed. Tian et al^28^ found that liver cells could express TSH receptor, and TSH could upregulate the expression of hepatic 3-hydroxy-3-methyl-glutaryl co-enzyme A reductase (a rate-limiting enzyme in cholesterol synthesis) by acting on the TSH receptor. TSH receptor could also play an important role in adipocyte differentiation and adipogenesis, resulting in obesity in mice and increasing BMI in humans.^29^ TSH could stimulate lipolysis in cultured adipocytes and elevate serum-free fatty acid levels in vivo.^30^ Besides, the involvement of leptin activation,^31^ visceral obesity^32^ and insulin resistance could also be relevant.^33^ Our study might shed some new light on the pathophysiological role of TSH on lipid in clinical perspective.

Gender difference on the association between TSH and lipids was the most important finding in our study. Males were more likely to suffer TSH-related dyslipidemia (Figs. 3 and 4), although females were more likely to have thyroid dysfunction (Table 2). Men demonstrated more protective effects of low TSH against hyperlipidemia, while females showed more detrimental effects of high TSH on hyperlipidemia (Tables 4 and 5). It is evidently recognized that male gender is associated with a markedly more proatherogenic lipid profile (higher LDL and lower HDL) than female gender. In addition, among women, menopause is associated with a significant shift toward an atherogenic lipid profile.^34–36^ Besides, there is also a sex dimorphism in the regional distribution of body fat. Evolutionary pressures predispose women to store excess fat in gluteal regions, while men are characterized by a greater accumulation of visceral adipose tissue.^37^ Visceral obesity is associated with increased atherogenic dyslipidemia.^38,39^ The traditional view of the above female advantage is thought to be due to the differences in the sex hormone milieu, in particular, the availability of estrogens and androgens.^40^ The endogenous estrogens have been reported to have a lowering effect on TC and LDL. And lipoprotein lipase is higher in women than in men.^41^ Now, it is thought that sexual dimorphism in lipid metabolism should be the result of a complex network of hormone action in combination with other, sex-specific, direct or indirect modulators of lipid metabolism.^35^

Besides gender, age should also be taken as a crucial factor for TSH-related dyslipidemia. Generally, aging is associated with deterioration of serum lipid profile.^42^ It is further recognized that women have less risk of atherosclerotic cardiovascular disease compared with men up until midlife (age 50–60), after which the gap begins to narrow, and hyperlipidemia in women even surpass those in men.^42,43^ Therefore, after menopause and beyond, lipid profile of women undergoes unfavorable changes, becoming worse than men.^44^ Our results were concordant with the previous studies. We found that young males, with the age of younger than 45 years, had significantly higher prevalence of hypercholesterolemia and high serum LDL than females. However, after menopause (older than 55), females had significantly higher prevalence than males. This crisscross pattern was also discovered in hypertriglyceridemia, yet the converging point was around 65 to 75 years of age. For the prevalence of low-serum HDL, males always showed higher prevalence than females.

Limitation of the study deserves comments. First, using a cross-sectional design, causality relationship could not be established. Prospective studies are necessary to truly determine their cause-and-effect relationship. Second, we applied stringent exclusion criteria to rule our relevant disease histories. However, since the annual health checkup policy is not good enough to cover the majority of Chinese, a number of the participants with various diseases might not be aware of them, which could be a confounding factor for our investigation. Third, the power of the present study could have been stronger if a multiethnic population and a much larger number of subjects were recruited.

In conclusion, we found that serum TSH levels were positively associated with the levels of TC, LDL, and TG, and the incidence of hyperlipidemia, these relationships were independent of thyroid hormones. Moreover, these associations were found to be stronger with increasing age. Males demonstrated more protective effects of low TSH against hyperlipidemia, while females showed more detrimental effects of high TSH on hyperlipidemia.
